# Using Boreholes as Windows into Groundwater Ecosystems

**DOI:** 10.1371/journal.pone.0070264

**Published:** 2013-07-31

**Authors:** James P. R. Sorensen, Louise Maurice, François K. Edwards, Daniel J. Lapworth, Daniel S. Read, Debbie Allen, Andrew S. Butcher, Lindsay K. Newbold, Barry R. Townsend, Peter J. Williams

**Affiliations:** 1 British Geological Survey, Wallingford, Oxon, United Kingdom; 2 Centre for Ecology and Hydrology, Wallingford, Oxon, United Kingdom; Utrecht University, Netherlands

## Abstract

Groundwater ecosystems remain poorly understood yet may provide ecosystem services, make a unique contribution to biodiversity and contain useful bio-indicators of water quality. Little is known about ecosystem variability, the distribution of invertebrates within aquifers, or how representative boreholes are of aquifers. We addressed these issues using borehole imaging and single borehole dilution tests to identify three potential aquifer habitats (fractures, fissures or conduits) intercepted by two Chalk boreholes at different depths beneath the surface (34 to 98 m). These habitats were characterised by sampling the invertebrates, microbiology and hydrochemistry using a packer system to isolate them. Samples were taken with progressively increasing pumped volume to assess differences between borehole and aquifer communities. The study provides a new conceptual framework to infer the origin of water, invertebrates and microbes sampled from boreholes. It demonstrates that pumping 5 m^3^ at 0.4–1.8 l/sec was sufficient to entrain invertebrates from five to tens of metres into the aquifer during these packer tests. Invertebrates and bacteria were more abundant in the boreholes than in the aquifer, with associated water chemistry variations indicating that boreholes act as sites of enhanced biogeochemical cycling. There was some variability in invertebrate abundance and bacterial community structure between habitats, indicating ecological heterogeneity within the aquifer. However, invertebrates were captured in all aquifer samples, and bacterial abundance, major ion chemistry and dissolved oxygen remained similar. Therefore the study demonstrates that in the Chalk, ecosystems comprising bacteria and invertebrates extend from around the water table to 70 m below it. Hydrogeological techniques provide excellent scope for tackling outstanding questions in groundwater ecology, provided an appropriate conceptual hydrogeological understanding is applied.

## Introduction

Groundwater ecosystems harbour invertebrate macro- and meio-fauna [1] and microorganisms [2]. Collectively, these may contribute to important ecosystem services such as biogeochemical cycling, pollutant attenuation, and maintaining open flow paths [3], [4]. Moreover, stygobitic invertebrates (obligate groundwater species) provide an important, but often overlooked, contribution to biodiversity [1], and are potential water quality indicators alongside microorganisms [5].

The absence of light in these ecosystems results in physiological adaptations and simple food webs dependent upon organic matter derived from the surface [6], [7]. However, a large proportion of this organic matter is biodegraded before reaching groundwater resulting in low and bio-limiting concentrations of dissolved organic carbon (DOC), nutrients, and trace elements [8], [9]. Therefore groundwater invertebrates have adapted: being slow-growing, having a slow metabolism, being long-lived, and having few young [1], [6]. Bacteria can respond to the poorly productive environmental conditions in groundwater by slowing their growth rates, and taking up resources at low concentrations [10], and may display reduced activity [11]. Invertebrate and microbial communities are naturally interlinked: invertebrates predate on bacteria but also assist their activity, e.g. via the breakdown of large particulate matter and generating nutrients via excretion or death [7]. Dissolved oxygen is an important control of subsurface ecology, for example macro-crustaceans are often tolerant of low oxygen concentrations, but anoxia for 2–3 days is fatal [12]. Aerobic or anaerobic conditions are also key in determining the type of microbial community present [10].

Groundwater ecosystems remain poorly understood due to the inaccessibility of the subsurface habitat that constrains both spatial and temporal sampling resolution [13]. Boreholes provide the only suitable sampling window into deeper non-karstic aquifers, and are commonly used for investigating invertebrates [14], [15] and microorganisms [16], [17]. Invertebrates within boreholes can be collected using nets, pumps, or traps [18], [19], but these samples are integrated over the water column and therefore the origin of the fauna within the aquifer is unknown. Furthermore, invertebrates may be concentrated in boreholes due to the accumulation of sediment and organic matter [20].

Characterising the distribution and abundance of invertebrates within aquifers, and understanding how representative a sampled borehole community is, are fundamental to understanding their potential contribution to ecosystem services and for conserving their biodiversity [21]; yet these issues remain unresolved. In this study, we addressed these questions by sampling water chemistry, and bacterial and invertebrate communities in isolated intervals at varied depths beneath the surface within two boreholes.

Our study was undertaken in the Cretaceous Chalk, which is the main source of freshwater in north-west Europe [22] and a habitat for invertebrate stygofauna in the UK [23]. The Chalk is a white limestone, composed of >98% calcium carbonate, with small-scale karst features [24]. The matrix has high porosity but low permeability. The high permeability is provided by fractures, fissures (fractures enlarged by dissolution which retain similar geometry) and conduits (tubular or rectangular voids). These provide the sole habitat for invertebrates because pores between mineral grains in the matrix are too small to access.

We used hydrogeological techniques (single borehole dilution tests and borehole imaging) to identify fractures, fissures or conduits within the aquifer that were intercepted by the boreholes. These habitats were isolated using packers (inflatable plugs), which are an established approach for investigating vertical differences in aquifer permeability [25], [26] and hydrochemistry [27], [28]. Previous studies have also used packers to investigate microbiological communities [29], and in shallow alluvial aquifers to examine invertebrate communities [30], [31], but not to explore the ecosystem collectively.

The study demonstrates a new method of investigating local habitats within groundwater ecosystems by combining a variety of hydrogeological and biological techniques with a new conceptual understanding of the origin of water, invertebrates and microbes in pumped samples. The use of packers enables biological and chemical samples to be obtained from targeted habitats within the aquifer. However, the study demonstrates the need to sample large volumes at a high pumping rate to obtain samples representative of the aquifer. Within this hydrogeological framework our study found that borehole water had more invertebrates, higher bacterial counts, and a different chemistry compared to aquifer water. Moreover there was variability in microorganisms and invertebrates within habitats at different depths illustrating ecological variability within the aquifer. A groundwater ecosystem comprising bacteria and stygobitic invertebrates is shown to extend from the water table to at least 70 m below it in the Chalk water supply aquifer.

## Methodology

### Overview

Packer testing was undertaken in two Chalk boreholes in which permeable features were identified during previous studies [25], [32], [33], [34]. In each borehole three potential aquifer habitats were selected at varied depths. These were isolated using inflatable packers and pumped to sample for stygobitic invertebrates, microbiology and hydrochemistry. Pressure changes within the interval were monitored during pumping to determine permeability.

### Borehole Setting

The selected boreholes were drilled into the Chalk aquifer in Berkshire, UK, in 2002. Beche Park Wood (BPW) penetrates 2 m of the Clay-with-Flints Formation overlying the Chalk, whilst Trumpletts Farm Borehole A (TFM) is located directly on unconcealed Chalk (Table 1). The boreholes are 2 to 2.5 km away from the nearest surface watercourse, and are open hole below the steel casing allowing visual imaging of the wall and enabling invertebrates to enter unhindered. TFM and BPW were accessed with the permission of D. Brown and Yattendon Estates, respectively.

**Table 1:** Borehole study sites.

BH	Latitude	Longitude	Elevation(m asl)	Land cover	Steel casing depth(m bd)	Depth(m bd)	Drilled diameter (mm)	WL range(m bd)	Water volume in BH (m^3^)
BPW	51.4919	−1.1977	143	Woodland	7	98	150	62.5–79.0	0.4
TFM	51.4720	−1.2629	108	Grass	18	100	150	13.5–23.5	1.8

Notes: BH is borehole; m aod is metres above sea level; m bd is metres below datum; datum is ground level at BPW and top of casing at TFM; water level (WL) range from continuous data between January 2003 and March 2012.

### Habitat Identification using Hydrogeological Techniques

In each borehole three aquifer habitats were selected using calipers, borehole imaging and single borehole dilution tests (SBDTs). Calipers consist of multiple prongs that measure the borehole diameter from a centralised point and produce logs of diameter variations with depth. Increases in borehole diameter beyond the drilled diameter may be indicative of fractures. Borehole imaging was conducted using MakiVion or a Geovista™ Optical Televiewer, which produce an unwrapped 2D image of the borehole wall. This allows visual identification of fractures, and indicates whether the fracture is horizontal or inclined, its aperture, and whether it has been enlarged by dissolution processes to form fissures or more circular shaped conduits. These techniques identify potential aquifer habitats intersected by the borehole, but do not demonstrate whether the feature is actively flowing or not.

SBDTs identify flow within and across boreholes enabling the identification of actively flowing features. They involve introducing a tracer into a borehole (salt in this instance) and obtaining profiles of tracer concentration with depth to monitor the tracer dilution. The characteristics of the dilution with depth over time can indicate whether the water flows across the borehole or moves vertically and out via another identifiable feature, and can give indicative estimates of groundwater residence time in the borehole [35]. Groundwater residence time was between 7 and 9 hours in TFM, whilst in BPW it was less than an hour above 87 m bd (below datum) and more than three months below 89.5 m bd. Details of horizontal and vertical flows identified from the SBDTs are provided in supporting information (Figure S1 in File S1).

### Packer Intervals

**Figure 1:**
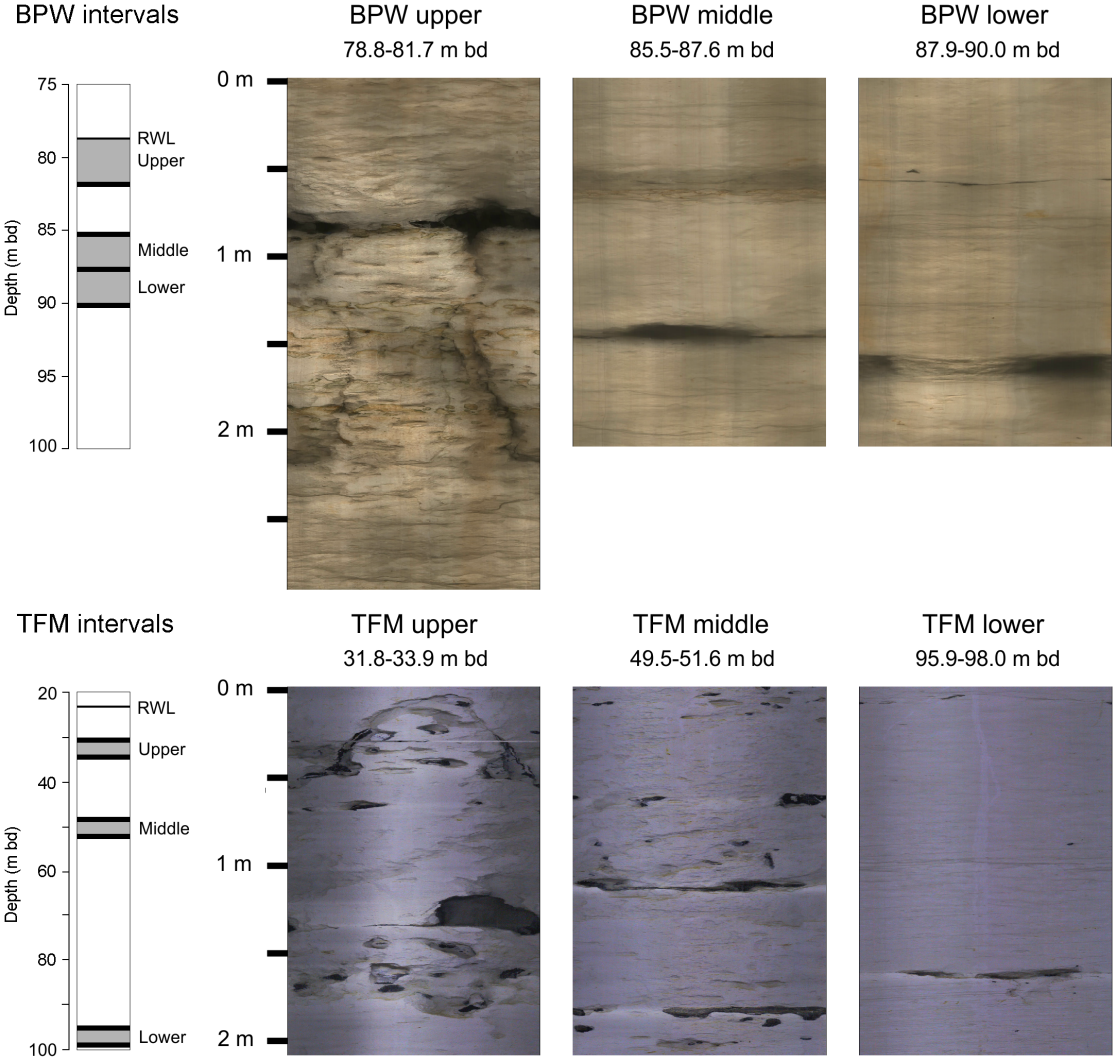
Unwrapped 360° optical images of packer intervals with horizontal exaggeration of X10. RWL is rest water level; m bd is metres below datum; datum is ground level at BPW and top of casing at TFM.

Inflatable packers were used to temporarily isolate three aquifer habitats intersected in each borehole. The selected intervals were spaced as far apart as possible to investigate ecological and hydrochemical differences with depth (Figure 1). The features comprised fractures, often solutionally enhanced, and conduits (Table 2). The interval length between the packers was 2.1 m, except for BPW upper where a single packer was placed 2.9 m below the water table to isolate an interval between the water table and the packer.

**Table 2:** Features in packer intervals.

Interval	Feature	Estimated aperture (m)	Location below top ofinterval (m)
BPW upper	solutionally enhanced bedding plane at top of sub-vertical fracture	0.07	0.8 to 2.1
BPW middle	solutionally enhanced fracture	0.07	1.5
BPW lower	conduit	0.12	1.6
TFM upper	conduit situated on a horizontal bedding plane	0.20	1.4
TFM middle	solutional fracture adjacent to flints	0.05	1.0
TFM lower	fracture with multiple solutionally enhanced openings	0.03	1.6

### Field Sampling Procedure

The packers were positioned around the fracture where the borehole walls were of most regular diameter (using the caliper log) to ensure a good seal. Packers were inflated with nitrogen gas to five bars above the hydraulic head to ensure the interval was sealed, e.g. if the packers were 50 metres below the water level, the inflation pressure was 10 bars. The interval was pumped with a Grundfos SQ 5–70 submersible pump with a maximum flow rate of 7 m^3^/hr, with large holes cut in the intake to minimise damage to invertebrates. During each interval test a total of 5 m^3^ was abstracted, which was equivalent to purging the interval 65–130 times.

To enable calculation of hydraulic conductivity (permeability of substrate to water), hydraulic head change within the interval was monitored with a pressure transducer during pumping and the discharge was measured with a flow meter. The hydraulic head of isolated intervals is generally different to that of the borehole water. Therefore a change in hydraulic head following packer inflation indicates that the packers are fully sealed and there is no leakage around the outside of the packers. Hydraulic conductivity was calculated according to equation 1, a simplification of Hvorslev [36]. Where: K is hydraulic conductivity, l is the length of the interval, r is the radius of the interval from the caliper log, Q is the abstraction rate and H is the change in hydraulic head during pumping.
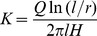
(1)


All abstracted groundwater was passed through a sample net and filter (63 µm) to capture invertebrates. The net and filter were changed after the following volumes had been pumped from each interval: 0.10, 0.25, 0.50, 0.75, 1.00, 1.50, 2.00, 2.50, 3.00, 3.50, 4.00, 4.50 and 5.00 m^3^. This enabled assessment of how the abundance and diversity of invertebrates changed with volume of water abstracted from the interval. All nets and filters were thoroughly washed down between each sample and their contents preserved in 4% formaldehyde.

Ten litre samples for microbiological analysis were taken immediately, and after 2.5 m^3^ and 5.0 m^3^. The first sample was representative of borehole water and the subsequent samples were aquifer water after the interval had been well purged. Each sample was split between two sterilised 5 L polycarbonate bottles, transported back to the lab, stored at 4°C in the dark, and processed within 24 h of collection. Samples for hydrochemistry were collected immediately after each microbiology sample. Four separate 60 mL samples were collected in HDPE plastic bottles: filtered for metals, anions including soluble reactive phosphorus (SRP) and ammonium, total dissolved phosphorus (TDP) and dissolved organic carbon (DOC), and unfiltered for total phosphorous (TP). The split for cations was acidified by the addition of 1% by volume concentrated AristaR grade HNO_3_, and the split for TDP and TP was pre-treated with 0.45 g of potassium peroxodisulfate and then immediately treated with sulphuric acid on return to the laboratory. Specific Electrical Conductivity (SEC), pH, and dissolved oxygen (DO) were measured onsite using an Aquaread™ Aquameter™ 200 calibrated daily. Alkalinity was also determined onsite by micro-titration.

When the packer testing was completed, a net sample was taken from the borehole to compare to the pumped interval samples. A weighted sample net (63 µm) was lowered to the bottom of the borehole, moved to agitate the sediments and then raised through the water column to the surface. This was undertaken three times and the contents of net and filter were preserved in 4% formaldehyde.

### Laboratory Procedures

#### Invertebrates

All samples were washed through a 100 µm sieve and sorted under a Zeiss Stemi 2000-C binocular microscope for invertebrates, which were enumerated. Crustaceans belonging to the Niphargidae family were identified to species level where possible using Gledhil et al. [37] and Knight and Gledhill [38]. Specimens which could not be identified to species were classified as *Niphargus* sp. though some smaller specimens where recorded as Niphargidae indet., if the *Microniphargus* genus could not be discounted. Nematoda were not identified further, and Oligochaeta were identified to family level using Brinkhurst and Jamieson [39].

Several specimens were damaged by the pump, leaving identifiable and unidentifiable pieces. Identifiable pieces contained one or more of the following features attached to a body segment: telson, gnathopods, or mandible palp. Where possible, body pieces within a sample were matched by size and shape to individual animals. When pieces clearly did not belong to the individuals in the sample and could not be matched to one another, they were counted as a separate individual thus assuming that some parts of the body were lost during sampling. Whole Niphargidae were also measured to the nearest 0.1 mm using an ocular graticule, along a straight line running from the base of the telson to the base of the antennae.

#### Hydrochemistry

Major anions were analysed by Dionex™ liquid chromatography on filtered (0.45 µm) samples. Metals were analysed by ICP-OES. Dissolved organic carbon (DOC) analysis was carried out using a Thermalox™ C analyser after acidification and sparging. P analysis was carried out within a week of sampling using different methods to determine different fractions. Soluble reactive P (SRP) is a measure of the inorganic monomeric and easily-hydrolysable phosphorus in the sample that has been filtered through a 0.45 µm filter [40], [41]. Total phosphorus (TP) is the combination of total dissolved P and particulate P (>0.45 µm) [42]. Total dissolved P (TDP) was determined after filtration through a 0.45 µm cellulose filter [42]. The dissolved hydrolysable P (DHP), polymeric and organic P, was calculated by difference.

#### Microbiology

One ml of water from each 10 L sample was fixed in 2% final concentration of particle-free formaldehyde (made fresh from molecular grade paraformaldehyde) and stored at −20°C until analysis. The remaining ≃10 L water sample (combining both 5 L polycarbonate samples) was sequentially filtered through a 2.7 µm GF/D prefilter (Whatman, UK) and then through an inline 0.22 µm Sterivex -GV Sterile Vented Filter Unit (Millipore, UK) using a peristaltic pump. Most free living bacterial cells should pass through the 2.7 µm filter and be caught on the 0.22 µm filter, thus representing the planktonic community. Any cells attached to the larger particulate matter caught in the 2.7 µm filter are assumed to represent the particle-associated bacterial community, although the separation of these two communities is not expected to be exact due to the dislodging of particle attached cells during the filtering process. Filters containing bacterial biomass were immediately frozen at −80°C for later analysis.

For bacterial cell counts, a 0.5 ml subsample of groundwater from each fraction and pumping time was stained using x1 final concentration of SYBR Green I stain (Invitrogen, UK). Samples were left to stain at room temperature for 10 minutes in the dark before analysis. A calibrated suspension of 6 µm polystyrene microspheres (Invitrogen, UK) was added to each sample and used to quantify cell numbers relative to the volume analysed. Stained bacterial cells were counted on a Beckman-Coulter Gallios flow cytometer using a 488 nm laser for excitation. Cell populations were distinguished using Side Scatter (SSC) and Fluorescence channel 1 (FL1 − 525 nm ±15 nm).

For the molecular characterisation of bacterial populations, total nucleic acids were extracted from both the GF/D and Sterivex membrane filters (representing particle attached and free living bacterial cells) using the PowerSoil®-htp 96 Well Soil DNA Isolation Kit using standard manufacturer instructions (MO BIO Laboratories, Inc). Approximately 20–30 ng of purified template was used per PCR. For T-RFLP analysis, a ≈500 bp region of the 16S small subunit ribosomal RNA (SSU rRNA) was amplified using 6-FAM labelled forward primer 27F and reverse primer 536R [43]. Thermal cycling conditions, enzymatic digestions and nucleic acid cleanup were carried out as per Newbold *et al*. [44] Fluorescently labelled restriction fragments were analysed on an Applied Biosystems 3730 DNA sequencer and the sizes of restriction fragments were calculated. This was only performed on the TFM samples as it was not possible to obtain PCR quality DNA extracts from the BPW samples. Binning analysis was performed using Genemarker (Softgenetics).

### Data Analysis

#### Statistics

Data were grouped by pumped volume and Kruskal-Wallis and Wilcoxon (both nonparametric rank sum tests) were used to explore statistically significant differences in hydrochemistry and bacterial abundance. The relationship between invertebrate size and pumped volume was tested using Pearson’s correlation. All statistics were peformed using R (version 2.8).

Binned bacterial TRFLP community profiles were normalised to the total area under the peaks for each profile. The profiles were analysed in the multivariate program PAST [45] using the Non Metric Multidimensional Scaling (NMDS) algorithm and a Bray-Curtis distance matrix. A one-way ANOSIM in PAST was used to test for significant differences between community structure between packer intervals and pumping intervals.

#### Theoretical sampling distances and velocities

The induced velocity within the borehole was calculated by dividing the pumping rate by the cross-sectional area (assuming a constant cross sectional area determined by averaging the diameters measured by the calipers). This flow is upwards towards the pump, due to its positioning within the packer system.

The distance into the aquifer that was sampled during pumping was calculated by using the pumping rate, and the void geometries and sizes observed in the borehole images; whilst the induced groundwater velocities at given distances from the borehole were calculated by dividing the pumping rate by the cross-sectional area of the flow face (see supporting information for more detail). These estimates assume constant void geometry extending infinitely beyond the borehole and horizontal radial flow.

## Results

### Hydrogeology and Groundwater Velocities

Hydraulic conductivity of the tested intervals varied between <1 and 486 m/d, and decreased with depth (Table 3).

**Table 3:** Interval hydraulic conductivities and theoretical distances sampled and velocities induced.

Interval	Q (L/sec)	K (m/d)	Intervalvolume (m^3^)	Upward velocityin BH (m/sec)	Distance fromBH sampled (m)	Velocity (m/sec)		
						Entry	1 m	5 m
BPW upper	1.12	486	0.065	0.05	5	0.34	0.02	0.005
BPW middle	0.77	41	0.041	0.02	5	0.23	0.02	0.003
BPW lower	0.44	1	0.039	0.04	22000	3.90	3.90	3.90
TFM upper	1.79	−	0.076	0.05	8000	5.70	5.70	5.70
TFM middle	1.73	286	0.050	0.07	6	0.73	0.05	0.01
TFM lower	1.17	0.6	0.039	0.06	7	0.83	0.06	0.01

Note: No head data are available to estimate permeability for TFM upper as the transducer failed; BH is borehole.

The volume of the isolated interval within the borehole varied between 0.04 and 0.07 m^3^. Induced groundwater velocities within the isolated borehole water column were between 0.05–0.07 m/sec at TFM and 0.02–0.05 m/sec at BPW (Table 3). The theoretical distance from the borehole from which aquifer water was drawn during pumping varied between 5 and 7 m for fractures and several kilometres for conduits (Table 3). The theoretical velocity of water as it entered the borehole was between 0.2 and 5.7 m/sec. These velocities dropped rapidly within fractures to 0.01 m/sec or less within 5 m of the borehole, but remained constant in the conduits in TFM upper and BPW lower (Table 3).

### Hydrochemistry

**Figure 2:**
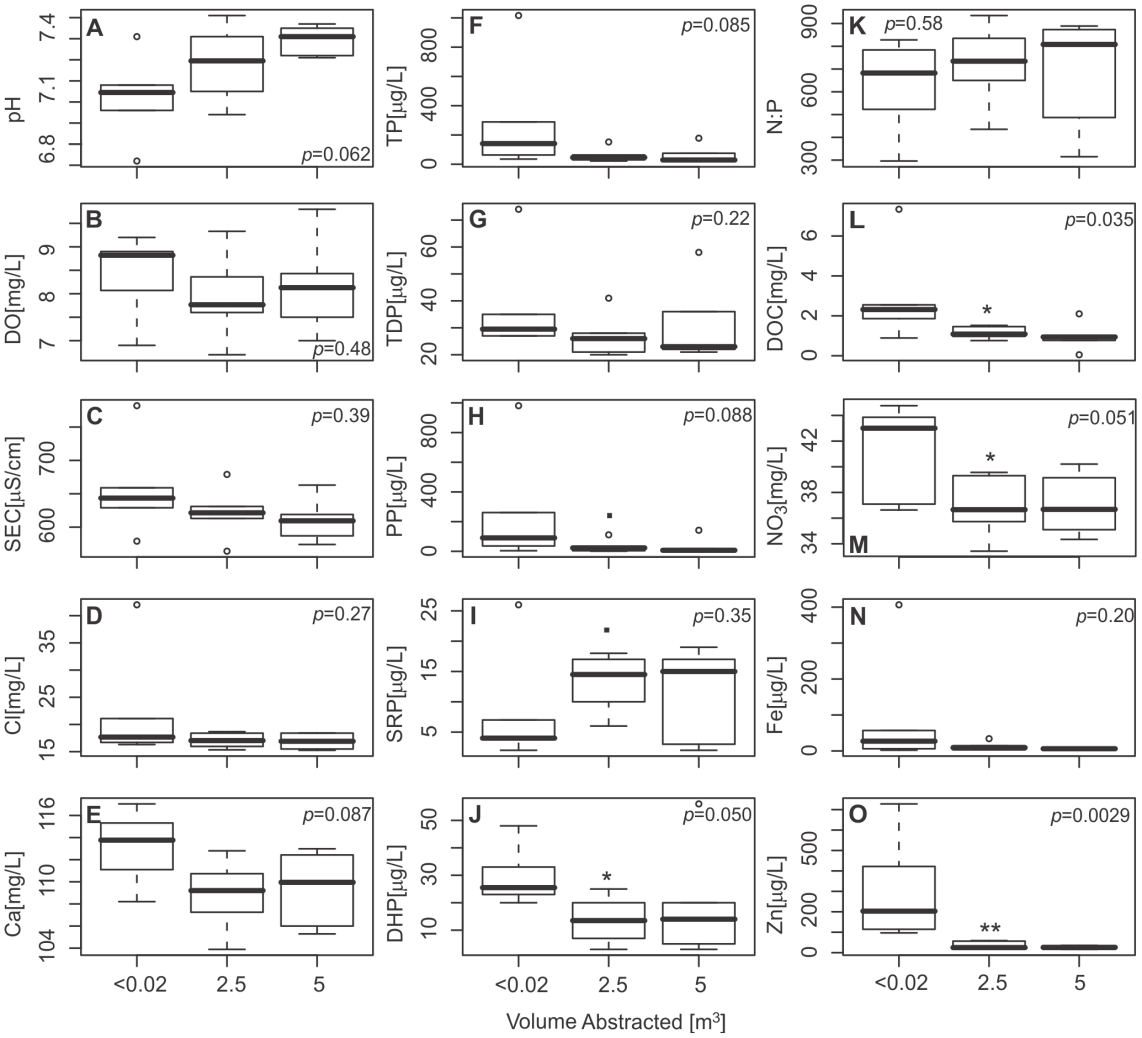
Grouped summary chemistry results. A) pH, B) DO, C) SEC, D)Cl, E)Ca, F)TP, G)TDP, H)PP, I)SRP, J)DHP, K) N:P(molar), L)DOC, M)NO_3_, N)Fe and O)Zn. Box = interquartile range, horizontal bar = median value, range of whiskers = ±1.5x interquartile range, outlier values are displayed = values that fall outside the range of whiskers. P values calculated using Kruskal-Wallis test. Wilcoxon rank tests were used to investigate differences between two pairs of groups, significance denoted as follow: **0.01, *0.05, ^▪^0.1.

Figure 2 shows that there are water chemistry differences between borehole water (samples taken after less than 0.02 m^3^ had been abstracted), and aquifer water (samples after 2.5 and 5 m^3^ had been abstracted). Within the borehole, there is a general trend of lower pH and SRP, and an enrichment of DO, SEC, Ca, all other nutrient species, and trace metals. However, the difference between borehole and aquifer water for pH, DO, SEC, Ca and Cl is not statistically significant (α = 0.05). Significant differences (α = 0.05) are observed for DHP, DOC, NO_3_, as well as some trace elements such as Zn and Cu. Changes in SRP and PP are significant at the (α = 0.1) level. There are no significant differences between the two aquifer water samples implying that all the borehole water had been flushed out of the system and fully equilibrated groundwater was being sampled after 2.5 and 5 m^3^.

**Figure 3:**
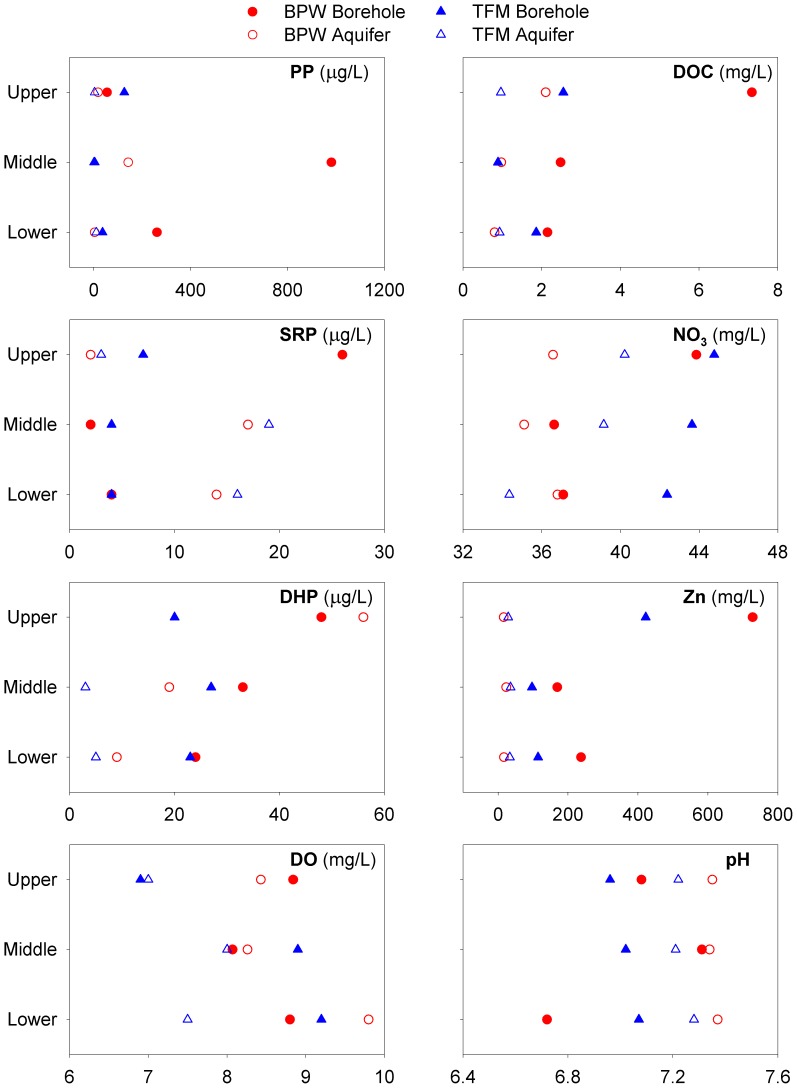
Changes in selected groundwater chemistry parameters for individual intervals. Aquifer water is sample after 5 m^3^ of pumping.

Figure 3 illustrates that there are no great differences in major ion hydrochemistry between intervals, although some differences in nutrients and trace elements do occur. Groundwater within all intervals is well-oxygenated in both the borehole and aquifer. pH values were generally near neutral in the borehole water but >7.2 after 5 m^3^ had been pumped in all intervals.

For most parameters, there is generally very little variation in water chemistry with depth in these Chalk boreholes. However at BPW, DOC and DHP decrease with depth in both borehole and aquifer water; whilst at TFM, NO_3_ decreases with depth (Figure 3). In both boreholes SRP is greater within the borehole in the upper interval. Furthermore, Zn concentrations are considerably higher within the borehole water of both upper intervals (see Table S1 in File S1 for full hydrochemical dataset).

### Microbiology

#### Bacterial cell counts

**Figure 4:**
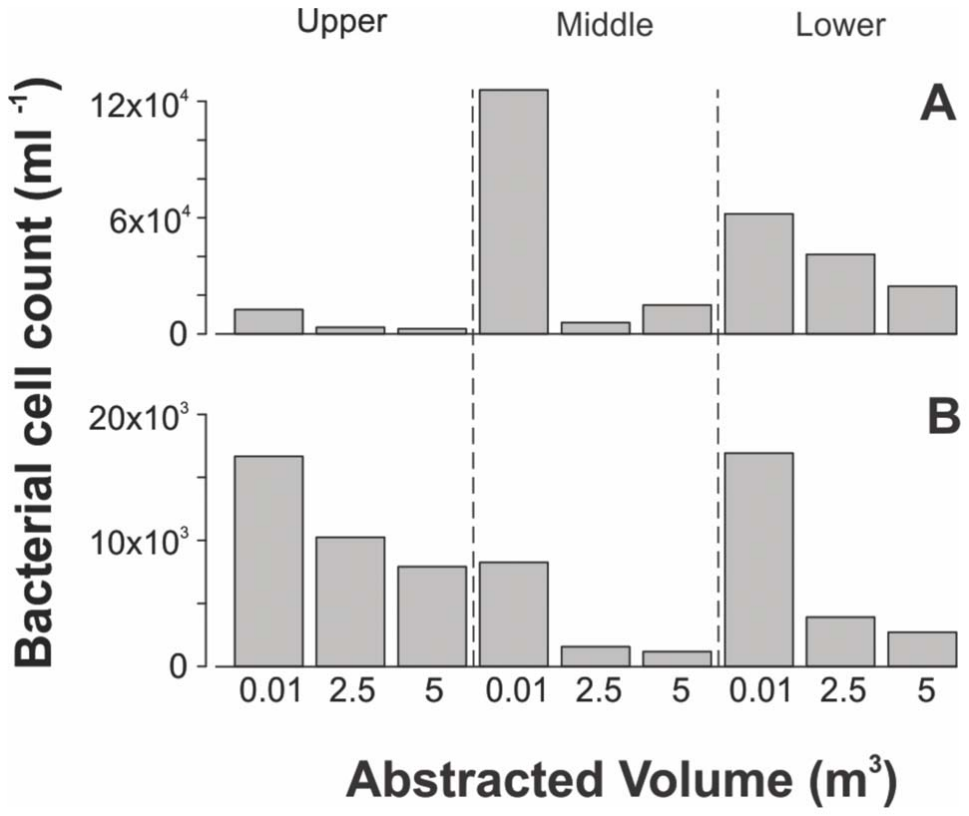
Bacteria counts by flow cytometry in intervals (A) BPW and (B) TFM.

Bacterial abundance, as determined by flow cytometry counts, was significantly greater within the borehole water than the aquifer water (*p* = 0.04, Wilcoxon test,) (Figure 4). In five out of the six intervals, abundance is greater in the aquifer sample after 2.5 m^3^ than at 5 m^3^, although this is not statistically significant based on the limited data points. Overall bacterial abundance within aquifer water samples varied between 1.1×10^3^ and 4.1×10^4^, with highest counts generally concurrent with intervals with the highest counts within the borehole water.

There is no clear correlation between bacterial abundance and depth. In BPW greatest abundance is within the middle interval in the borehole and lower interval in the aquifer. At TFM the lower interval and upper interval have greatest abundance in the borehole and aquifer, respectively. BPW generally contained a greater abundance than TFM in both the borehole and aquifer.

#### Bacteria population molecular characterization

**Figure 5:**
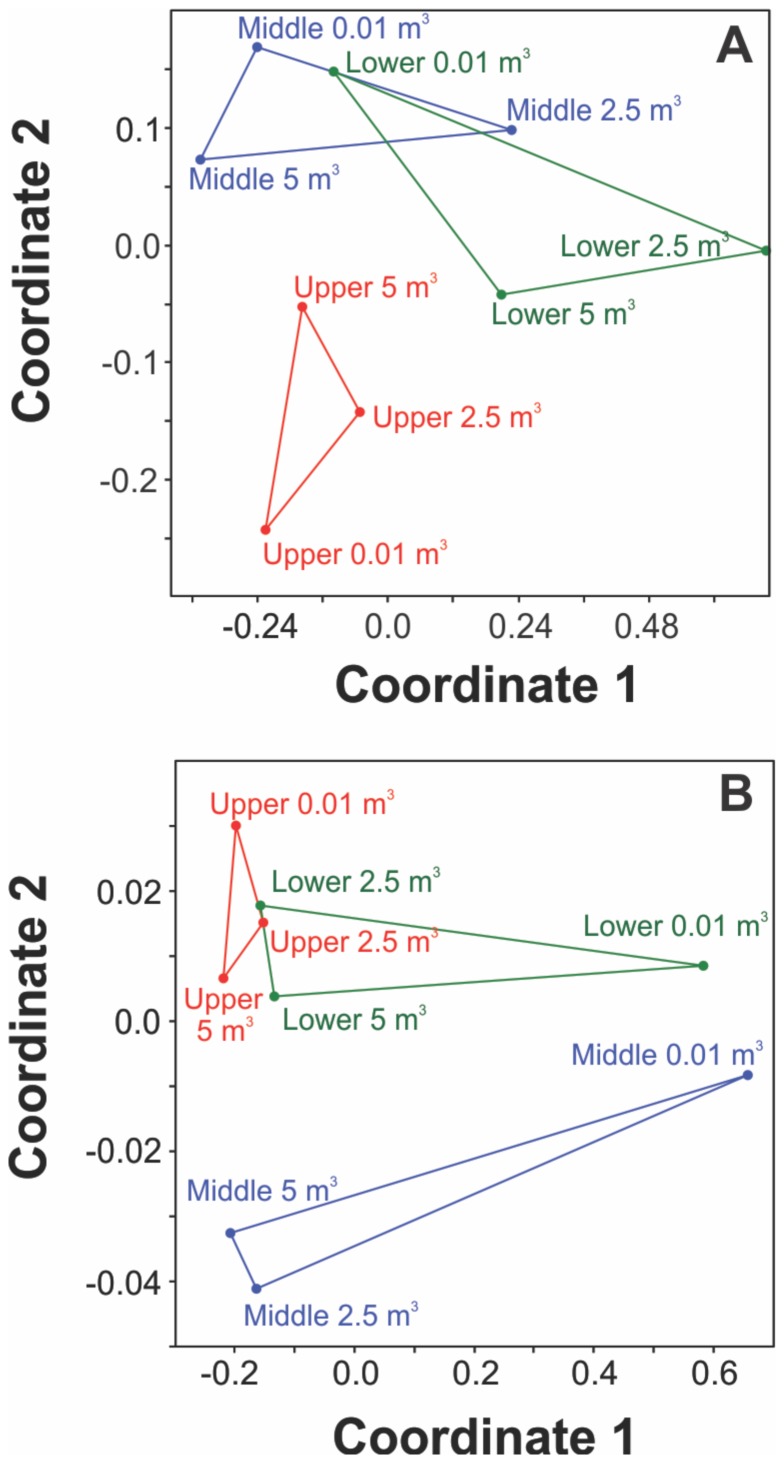
TRFLP profiles at TFM for (A) planktonic bacteria (B) particle associated bacteria. Non-Metric Multidimensional Scaling (NMDS) plot with solid lines illustrating the interval, but not statistical significance.

Terminal Restriction Fragment Length Polymorphism (TRFLP) of the bacterial communities from TFM show a visual separation in community structure in the NMDS plots (Figure 5). In the planktonic phase, the upper interval forms a distinct cluster away from the middle and lower intervals (Figure 5a). In the particle associated bacteria the middle interval forms a distinct, although less tightly clustered grouping (Figure 5b). In both the planktonic and particulate associated fractions the borehole samples (<0.01 m^3^) from the middle and lower fractions were similar, as determined by their proximity in the NMDS plot, and different from the aquifer water samples. None of the above observations are statistically significant, due to the limited number of data points (one-way ANOSIM).

### Invertebrates

Invertebrates were captured in all packer tested intervals, and predominantly comprised stygobitic Niphargidae that were present in 5 out of 6 intervals (Table 4). Most specimens were *Niphargus kochianus kochianus*, with *Microniphargus leruthi* also present in TFM. Where present, the numbers of Niphargidae ranged from 3 to 12. Additionally, two specimens of the Lumbriculidae family (Oligochaeta), one nematode worm and one specimen of the Enchytraeidae family (Oligochaeta) were collected from different intervals in BPW.

**Table 4:** Number of invertebrates in packer and net samples.

Interval	N. kochianus kochianus	M.leruthi	Niphargussp.	NiphargidaeIndet.	Lumbriculidae	Enchytraeidae	Nemotodes	Total
BPW upper	0	0	0	0	1	0	1	2
BPW middle	8	0	0	4	1	0	0	13
BPW lower	7	0	0	3	0	1	0	11
BPW nets	19	0	0	0	1	1	0	21
TFM upper	0	2	1	0	0	0	0	3
TFM middle	4	3	1	3	0	0	0	11
TFM lower	1	2	2	1	0	0	0	6
TFM nets	28	1	0	0	0	0	0	29

Note: No meio-fauna (including copepoda) were present in any samples.

*Niphargus kochianus kochianus* was also the most abundant species in the net samples from both boreholes. *Microniphargus Leruthi* was also identified in TFM, and specimens of Lumbriculidae and Enchytraeidae were captured in BPW. Therefore all taxa present in the net samples were also represented in the pumped interval samples, with the exception of nematodes. However, the net samples contained comparatively larger numbers of Niphargidae. At BPW, with a total water column of 0.4 m^3^, 19 specimens were collected in the three net samples whereas only 22 were collected from pumping a total of 15 m^3^ from three of the four potential habitats which intersect the borehole (Figure S1 in File S1). Furthermore, these net samples were collected after over a third of the borehole volume had been isolated, pumped and presumably emptied of fauna. At TFM, the same trend was observed, with 29 specimens collected from a water column of 1.8 m^3^ but only 20 individuals sampled from the 15 m^3^ pumped from the intervals.

**Figure 6:**
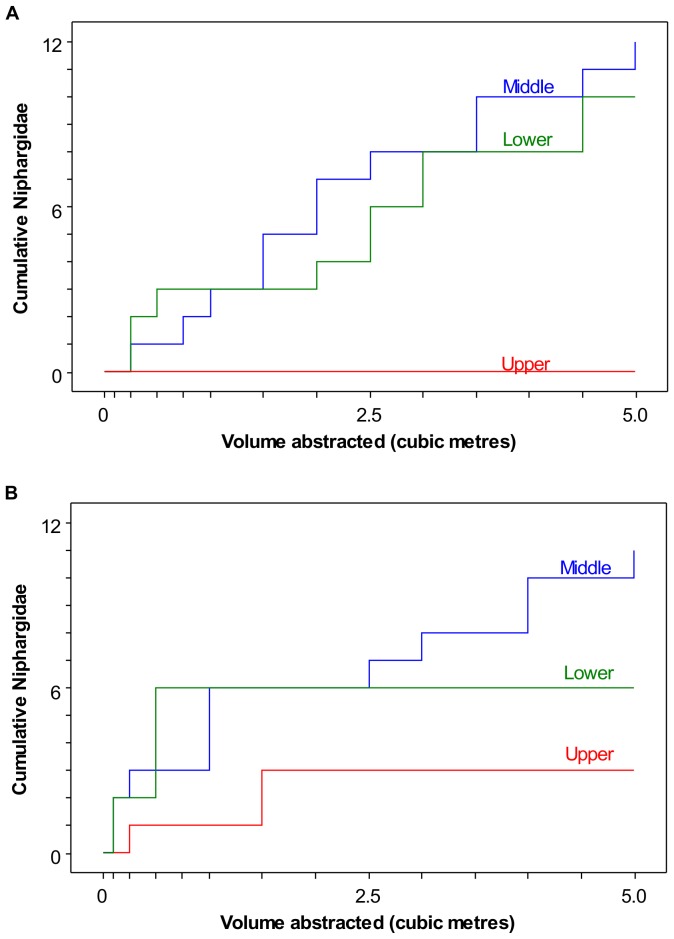
Cumulative Niphargidae captured with volume abstracted from each interval (A) BPW (B) TFM.

The Niphargidae were captured after different volumes of water had been pumped from the intervals (Figure 6). In three intervals Niphargidae continued to be collected progressively over time (BPW middle and lower, and TFM middle), whilst in 2 intervals (TFM lower and upper) they were only collected during the early part of the test (up to 0.5 and 1.5 m^3^). None were collected from BPW upper. There was no significant correlation (*p* = 0.62, n = 17, Pearson correlation) between size of individual Niphargidae and the pumped volume at which they were captured (see supporting information for data).

## Discussion

### Origin of Sampled Invertebrates

#### Theory

**Figure 7:**
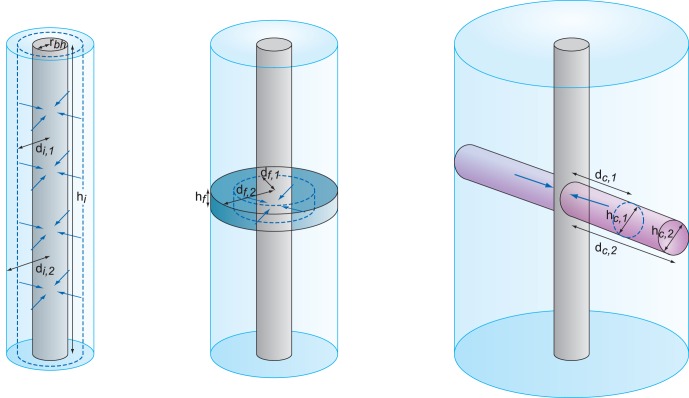
Flow to a borehole: intergranular aquifer, a single fracture, a single linear conduit. *d* is distance from borehole after volume (1), then greater volume (2) are abstracted, *h* is height of flow face, *r* is borehole radius.

The sampled distance away from a borehole and into the aquifer can be calculated for different types of aquifers by assuming horizontal flow and cylindrical geometry (Figure 7). In intergranular flow aquifers, e.g. sands and gravels, water is drawn to the borehole radially through connected pore space along the entire length tested; therefore only a small distance is sampled. In an isolated fracture, assuming a single planar feature of constant aperture, a larger distance is sampled as water flows through a smaller open flow face which increases in area away from the borehole. The greatest distance sampled would be when flow is through a single linear conduit as the flow face is relatively small and does not increase with distance from the borehole (see supporting information for more detail).

Differences in theoretical sampled distances for different aquifer flow systems using a range of realistic porosity and aperture values are shown in Table 5. Distances are shown for a total groundwater abstraction of 5 m^3^, and the 0.05 m^3^ suggested as a minimum for pumped invertebrate samples by Malard et al. [46]. These theoretical distances are associated with uncertainty, e.g. features may be interconnected (leading to overestimation of distances) or they may not be laterally extensive in multiple directions (leading to underestimation). Nevertheless, it is clear large volumes are needed to sample water beyond the immediate environs of the borehole in intergranular and fracture flow systems.

**Table 5:** Theoretical sampled distances from boreholes for different flow systems assuming an interval length of 2.1 m.

Flow system	Feature properties	Distance from borehole sampled (m)	
		Volume pumped after purging	
		0.05 m^3^	5 m^3^
Intergranular	25% porosity	0.2	1.7
	45% porosity	0.1	1.3
Fracture	2 mm aperture	2.8	28
	10 mm aperture	1.3	13
Conduit	25 mm radius	12.7	>1200
	100 mm radius	0.8	80

The likelihood of capturing invertebrates depends upon the velocity induced by pumping. It has been suggested that catastrophic drift for Niphargidae occurs above around 0.1 m/sec [47], based upon experiments on *Niphargus virei* and *Niphargus rhenorhodanensis* [48]. These studies also suggest that these species are rheophilic resulting in behavioural drift as they deliberately follow detectable currents, including those induced by pumping. However, we are not aware of any studies demonstrating the velocity required to induce behavioural drift in Niphargidae.

**Figure 8:**
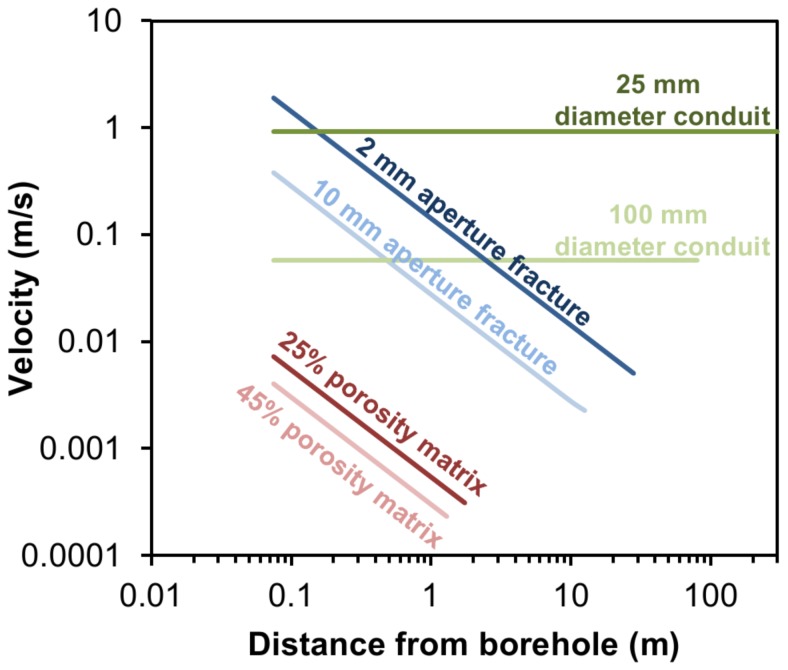
Theoretical maximum velocities with distance away from a borehole in different flow systems. Assuming a 150 mm diameter borehole, a 2.1 m length test interval, a pumping rate of 1.79 L/sec, and a total of 5 m^3^ is abstracted.

The induced velocity at any given point in an aquifer or borehole can be calculated by dividing the pumping rate by the cross-sectional area of the flow face (see supporting information for more detail). Induced groundwater velocities vary depending on the aquifer type (Figure 8). Within a matrix system velocities are very low, and for matrix systems and fractures, decrease inversely proportionally with distance from the borehole, whilst they remain relatively high and constant in a linear conduit.

#### Application to this study

In our study, vertical flows within the borehole (0.02 to 0.07 m/s) were below that required for catastrophic drift (0.1 m/s), but were rapid for groundwater and their onset was sudden which is likely to favour the entrainment of Niphargidae. Entry velocities into the borehole were always >0.1 m/sec suggesting catastrophic drift was likely for invertebrates in close proximity to the borehole.

Within the intervals where a fracture was sampled, theoretical velocities (Table 3) suggest catastrophic drift was unlikely beyond a metre from the borehole. However this assumes a constant open aperture extending uniformly away from the borehole and in reality there are likely to be areas where the fracture is closed, reducing the rate at which velocities decline with distance from the borehole. Invertebrates from fractures are therefore likely to have originated from more than a metre away, although given the limited radial distance influenced by pumping 5 m^3^, catastrophic drift is unlikely to have occurred more than 5 m from the borehole.

Within TFM upper and BPW lower, where conduits were sampled, catastrophic drift was theoretically possible over distances of kilometres. However, in practice, a single isolated conduit is unlikely to persist over long distances in the Chalk. Instead, it is likely to form part of a dense connected network of different flowing features [32]. Therefore, catastrophic drift was likely to have been restricted to within tens of metres of the borehole.

We conclude that a sufficient volume was pumped at a high enough rate to capture invertebrates from the aquifer. The absence of fauna during later stages of pumping in TFM lower and upper suggests fauna originated from near the borehole. In other intervals where fauna were captured progressively with pumped volume they are likely to have originated from up to 10s of metres from the borehole. Nevertheless, it is uncertain whether at these distances we are sampling an undisturbed aquifer community, or if the community is affected by the proximity to the borehole.

#### Wider application and sampling implications

Our study demonstrates that large volumes and high pumping rates are needed to sample invertebrates beyond the confines of the borehole in intergranular and fracture flow systems. Using a low flow rate (a few L/min), e.g. Hahn and Matzke [20], will invoke negligible velocity through an intergranular aquifer and may not actually sample invertebrates from the aquifer. Furthermore, induced velocity at these low flow rates within a single isolated fracture will be low and will decrease below 0.01 m/sec within <1 m of the borehole. Therefore, any captured invertebrates are likely to be from the borehole or immediate proximity. Sampling long intervals will also invoke reduced velocities in the aquifer because the velocities will be divided over multiple fractures, or a longer vertical distance in intergranular aquifers.

The rapid decrease in velocity away from a borehole in intergranular and fracture systems could partially explain the non-linear decreases in collected fauna with volume observed by Boulton et al. [49]. Our study supports their conclusions that abundance should not be expressed as individuals per unit volume. Instead the time when an individual was captured during pumping should be reported, and using the framework presented here, it can be inferred whether it is likely that the specimen originated from the borehole or aquifer.

### Origin of Sampled Bacteria

Bacterial cell counts comprise truly planktonic bacteria, and entrained free and particle-associated bacteria that have been mobilised during pumping (Figure 4). Planktonic bacteria originate from the same place as the water in which they were sampled. However the origin of free and particle-associated bacteria entrained through the disturbance of sediments and shearing of cells from biofilms, within or attached to the borehole walls and fractures, is more uncertain. This will depend on the duration and rate of pumping, with higher pumping rates demonstrated to increase bacterial counts [50], [51].

Bacterial abundance was generally higher within the sample after 2.5 m^3^ than the sample after 5 m^3^ of pumping. This may be because the bacterial community in the 2.5 m^3^ sample was still affected by the presence of the borehole, despite the fact that 30–60 borehole volumes had been pumped and the water originated from several metres or tens of metres from the borehole. This may be because the high pumping rate continued to cause shearing of bacteria from biofilms within the borehole. Alternatively, the 2.5 m^3^ sample may comprise bacteria from the fractures and conduits in the vicinity of the borehole where bacterial communities may still be affected by the borehole presence and differ from those in the wider aquifer [50]. The latter suggestion is supported by evidence indicating a difference in community structure between the borehole water and aquifer water (Figure 5a), suggesting that the 2.5 m^3^ sample may comprise bacteria originating from the aquifer.

These results emphasise the importance of significant well purging for a representative understanding of bacterial abundance within the aquifer, as observed by Kwon et al. [50]. Purging three well volumes or until chemical parameters have stabilised, e.g. Roudnew et al [52], may be insufficient. In our study it is uncertain whether the 5 m^3^ sample was also influenced by the presence of the borehole. Consistent stable bacterial counts in two or more samples taken at later stages during pumping are needed to be certain that samples are from the aquifer.

### Comparison of Borehole and Aquifer Waters

Biological activity appears greater within the borehole, with significantly higher bacterial counts and numbers of invertebrates than in the surrounding groundwater. The borehole waters were also enriched in particulate and organic (complex) P sources as well as DOC and NO_3_, and depleted in SRP with respect to the regional aquifer water. This suggests that the borehole community utilises the bioavailable SRP supplied by the regional groundwater. The localised biological cycling of nutrients causes an increase in DOC, NO_3_, and more complex organic forms of P within the borehole due to the in-situ decay and breakdown of biological matter. This suggestion is also corroborated by the generally lower pH within the borehole waters.

The high NO_3_ concentrations compared to SRP and DOC, suggests the Chalk ecosystem is not likely to be limited by nitrate. It is not possible to determine whether P or DOC are the most limiting, because although SRP concentrations are very low, much of the detected DOC in aquifers is likely to be recalcitrant and not readily bioavailable [53].

The higher concentrations of trace elements (e.g. Fe, Zn Ni, Cu) within the borehole are likely to be due to corrosion and leaching of the borehole casing. This may affect the abundance and diversity of stygofauna and microorganisms within boreholes completed with steel casing. For example, a study suggested steel casing may be less favourable to bacteria than PVC [54], but Fe could also encourage iron-reducing bacteria. Indeed, our results suggest the structure of the bacterial community may differ between the borehole and aquifer, although we only obtained a small number of samples and therefore further investigation is needed.

### Vertical Extent and Variability of the Chalk Aquifer Habitat

There were small differences between investigated habitats in terms of invertebrate and bacteria abundance. Nevertheless, invertebrates were captured in all intervals, and the study shows that Niphargidae inhabit both conduits and solutional fissures within the Chalk. Furthermore, only minor hydrochemical variations were noted in nutrients and trace elements, with high dissolved oxygen and sufficient nutrients throughout. Our results show that in these boreholes there is very little variability in the water chemistry of fissures and conduits intercepted at different depths ranging from 34 to 98 m below the surface in the Chalk. Bacterial abundance varied by only an order of magnitude within the aquifer, with no consistent pattern with depth. A groundwater ecosystem comprising bacteria and stygofauna has been observed extending from the water table to 70 m below it. There is no apparent relationship between biological variables and permeability.

In the study area, beyond the vertical extents of the boreholes, the Chalk aquifer continues down for a further c.100 m. However, available habitat within the Chalk (like many aquifers) will become scarcer and increasingly isolated with depth, as fracture spacing increases and fracture aperture decreases [55]. Nevertheless, the chemistry of the water is likely to remain suitable with relatively high dissolved oxygen throughout the unconfined aquifer [56], although DOC [10] and nutrients may become increasingly depleted. It is uncertain how bacterial populations might change with depth, but reduced carbon and nutrients are likely to limit microbial activity to a greater extent and elsewhere authors have suggested bacterial abundance generally decreases with depth [52], [57]. Therefore the potential food supply for invertebrates may also decrease with depth.

### Applicability of Hydrogeological Techniques to Groundwater Ecosystem Studies

Borehole imaging and single borehole dilution tests can rapidly characterise the localised aquifer habitat intercepted by a borehole. They allow identification of fractures, fissures and conduits, indicate whether these are actively flowing, and allow estimates of water residence time within sections of a borehole.

When taking pumped samples from existing boreholes, there are uncertainties surrounding the origin of sampled invertebrates and microbiology within the aquifer, and disentangling borehole and aquifer communities remains challenging. Packer systems can be used to isolate and characterise individual habitats intercepted by a borehole, assess variability between these, and thus evaluate vertical differences within aquifers. Thus packers provide greatly improved knowledge on the origins of a pumped groundwater sample. Moreover, packers allow a small section to be isolated allowing a greater velocity to be induced within the aquifer to entrain organisms; hence a greater distance from a borehole can be sampled.

To ensure invertebrates are entrained from the aquifer at least several metres away from the borehole it is necessary to use as high a pumping rate as feasible (ideally >1L/sec) and sample several m^3^ of water. Separating aquifer and borehole communities could be achieved most easily by packer testing newly drilled boreholes, although it has been demonstrated that the drilling process can affect the groundwater bacterial community [54].

### Conclusions

We successfully identified and comprehensively sampled six local habitats at varied depths in two Chalk boreholes. Our study provides a new hydrogeological framework for understanding and investigating local habitats intersected by boreholes in aquifers. Combining different types of borehole data (images, calipers and single borehole dilution tests) with packer testing techniques enables individual habitats to be identified, characterised (in terms of void type and permeability) and investigated. We demonstrate a new sample methodology for obtaining near-simultaneous information on water chemistry, microbiological and invertebrate communities, thereby providing a means of investigating the entire ecosystem present within these localised habitats. The study demonstrates the advantages of using packer testing to investigate local variability in groundwater ecosystems. Furthermore we demonstrate how to distinguish borehole water from aquifer water, and provide a new conceptual understanding of where sampled water, invertebrates and microbial communities originate from during pumped sampling; which depends upon the pumping rate, borehole volume, and geometry of the voids present within the aquifer, as well as the entrainment characteristics of the organisms.

The results showed that there are significant differences between the borehole and aquifer water, suggesting that the boreholes are sites of enhanced biogeochemical cycling. Bacterial counts, invertebrate abundance and organic P, DOC and NO_3_ were all higher in the borehole than in the aquifer, whilst bioavailable SRP was lower in the borehole than the aquifer. There was also some evidence that the bacterial community structure differed between the borehole water and aquifer water.

There were some variations in the chemical and biological characteristics of the aquifer habitats sampled. In some intervals fauna were consistently collected during later stages of pumping, and our calculations and conceptual model suggest that they are likely to have occupied habitats five to tens of metres from the borehole. In contrast, in other intervals fauna were only captured early on in pumping indicating they did not inhabit the aquifer far beyond the confines of the borehole. There is also evidence of some variation in bacterial community structure between habitats within the aquifer, although bacterial abundance only differs by an order of magnitude. Major ion chemistry, including dissolved oxygen, remained similar, and the study shows that within the UK Chalk water supply aquifer, an ecosystem comprising bacteria and invertebrates extends from the water table to at least 70 m below it.

This study illustrates that sampling the borehole environment is not biologically or chemically representative of the aquifer. To sample the aquifer and entrain invertebrates from outside the borehole large volumes of water (m^3^) must be abstracted at a high pumping rate (>1 L/sec). In this study bacterial counts appeared different in samples collected after 2.5 and 5 m^3^ of pumping suggesting that the bacterial community sampled was influenced by the borehole even this late in the test. A greater volume would need to be pumped to obtain samples with similar counts indicating that the aquifer away from the influence of the borehole has been sampled.

Future studies are needed to build on these observations to improve our understanding of the location, diversity and abundance of biological communities within aquifers. This will enable a better understanding of their ecosystem services and enable better conservation efforts. Hydrogeological techniques could play a pivotal role in this future groundwater ecosystem research provided they are applied with suitable conceptual understanding.

## Supporting Information

File S1 Figure S1. SBDTs at (A) TFM and (B) BPW with borehole flow regimes and packer intervals in this study. Times refer to time (h: hours, d: days) after dilution and B is background; RWL is rest water level; U, M, L are upper, middle and lower intervals, respectively. Table S1. All hydrochemical data. Note: Number in interval name refers to when sample was taken during pumping; all forms of phosphate and nitrogen are total concentrations. Table S2. Sizes of whole captured invertebrates with pumped volume.
(ZIP)
