# Supplementary material for: Using Boreholes as Windows into Groundwater Ecosystems
Source: PLoS One. 2013 Jul 31;8(7):e70264. doi: 10.1371/journal.pone.0070264 (PMC3729555; doi:10.1371/journal.pone.0070264)
Supplement: File S1 — Figure S1. SBDTs at (A) TFM and (B) BPW with borehole flow regimes and packer intervals in this study. Times refer to time (h: hours, d: days) after dilution and B is background; RWL is rest water level; U, M, L are upper, middle and lower intervals, respectively. Table S1. All hydrochemical data. Note: Number in interval name refers to when sample was taken during pumping; all forms of phosphate and nitrogen are total concentrations. Table S2. Sizes of whole captured invertebrates with pumped volume. (ZIP) [file pone.0070264.s001.zip › Supporting information 31-05-13.docx]

**Supporting information**

Borehole flow regimes

Under ambient flow conditions, in boreholes that intercept fractures with different hydraulic head, water flows vertically up or down a borehole from fractures of higher hydraulic head to fractures of lower hydraulic head ([Church and Granato, 1996](#_ENREF_2); [Elci *et al.*, 2001](#_ENREF_3); [Shapiro, 2002](#_ENREF_6)). Previous studies indicate complex flow patterns within both boreholes used in this study; these are included as arrows on Figure S1, which also shows the results of the dilution tests that were used to identify flows. The flow patterns in Trumpletts A indicated in Figure S1 have been consistently demonstrated by borehole impeller flow measurements ([Butler *et al.*, 2009](#_ENREF_1)), uniform injection SBDTs ([Williams *et al.*, 2006](#_ENREF_7); [Maurice *et al.*, 2012](#_ENREF_5)), and point tracer injection SBDTs ([Maurice, 2009](#_ENREF_4)). Point tracer injections indicated upward vertical flow from an inflowing feature at 97.6 m below datum (m bd) to an outflowing feature at 34 m bd, with a decrease in the rate of upward vertical flow from c. 340 to c. 60 m/day caused by at an additional outflowing feature at 51.5 m bd. Other flowing features were identified at 28.5 and 81.3 m bd (Figure S1). Under ambient conditions, dilution of tracer from uniform injection SBDTs (indicative of water residence time in the borehole) took approximately 7 to 9 hours.

Single borehole dilution testing at Beche Park Wood ([Maurice, 2009](#_ENREF_4)) indicated flowing features at 76.9, 79.6, 87 and 89.5 m bd and a very slow flowing feature towards the base of the borehole (Figure S1). Vertical flows are less certain because point tracer injections were not carried out. However, the uniform injection test results suggest there may be upward flow from 79.6 to 76.9 m bd and downward flow from 79.6 m bd to the bottom, with decreases in flow rates caused by outflowing features at 87 and 89.5 m bd. Dilution times varied in different sections of the borehole from less than an hour in the section between 79.6 and 87 m bd to more than three months in the slow flowing section between 89.5 m bd and the base.

These results explain why water chemistry varies between samples taken from different depths within the borehole. The boreholes intercept water in several fractures with different chemistry which are mixed together within the borehole by the vertical flows. Each packer interval intercepts a different inflowing fracture, or a different mixture of water from more than one fracture. In addition residence times within BPW vary substantially from less than an hour to many months which could also create differences in water chemistry within the borehole.

Hydrochemical data

Table S1 All hydrochemical data

Invertebrate size data

Table S2 Sizes of whole captured invertebrates with pumped volume

Estimating distance from a borehole sampled for groundwater

The distance away from a borehole that is sampled when groundwater is pumped from the aquifer can be estimated for intergranular aquifers by equation 1, through a single fracture by equation 2, and through a linear conduit by equation 3. If multiple flowing features are present then water will be preferentially drawn through the more permeable horizon(s), and these estimates become more uncertain.

 (1)

$d_{(i)}= \sqrt{\frac{(v/\varphi)}{\pi h_{(i)}}}$Where: *d* is distance from borehole, *v* is volume of water abstracted, $\varphi$ is porosity, *h* is height of the flow face.

$d_{(f)}= \sqrt{\frac{v}{\pi h_{(f)}}}$ (2)

$d_{(c)}=0.5 \times\frac{V}{\pi(0.5 \times h_{(c)}^{2})}$ (3)

These equations assume regular cylindrical geometry, horizontal radial flow, the diameter of the borehole is infinitesimally small; and no boundary conditions, no vertical permeability differences within the intergranular tested section, no interconnectivity between fractures/conduits, and no contribution from matrix waters in fracture and conduit systems.

Estimating induced groundwater velocity at a specific distance from a borehole

The induced velocity by pumping within a flowing feature at any distance from a borehole can be estimated by dividing the abstraction rate by the cross-section area of the flow face. Therefore, velocity can be estimated at a given distance in an intergranular aquifer using equation 4, through a single fracture by equation 5, and through a single linear conduit by equation 6.

$V_{(i)}= \frac{Q}{2\pi{(d}_{(i)}{+ r_{bh})h}_{(i)}\varphi}$ (4)

Where: *Q* is pumping rate

$V_{(f)}= \frac{Q}{2\pi{(d}_{(f)}{+ r_{bh})h}_{(f)}}$ (5)

$V_{c}=\frac{Q}{\pi{{(0.5 \times h}_{c}^{2})}}$ (6)

The assumptions are the same as those for equations 1-3.

References

Butler A, Mathias S, Gallagher A, Peach D, Williams A. 2009. Analysis of flow processes in fractured chalk under pumped and ambient conditions (UK). Hydrogeol. J., **17**: 1849-1858.

Church PE, Granato GE. 1996. Bias in ground-water data caused by well-bore flow in long-screen wells. Ground Water, **34**: 262-273.

Elci A, Molz FJ, Waldrop WR. 2001. Implications of observed and simulated ambient flow in monitoring wells. Ground Water, **39**: 853-862.

Maurice LD. 2009. Investigations of rapid groundwater flow and karst in the Chalk. University College London.

Maurice LD, Atkinson TC, Barker JA, Williams AT, Gallagher AJ. 2012. The nature and distribution of flowing features in a weakly karstified porous limestone aquifer. Journal of Hydrology, **438–439**: 3-15.

Shapiro AM. 2002. Cautions and suggestions for geochemical sampling in fractured rock. Ground Water Monitoring and Remediation, **22**: 151-164.

Williams A, Bloomfield J, Griffiths K, Butler A. 2006. Characterising the vertical variations in hydraulic conductivity within the Chalk aquifer. Journal of Hydrology, **330**: 53-62.
