# Supplementary material for: Using Boreholes as Windows into Groundwater Ecosystems
Source: PLoS One. 2013 Jul 31;8(7):e70264. doi: 10.1371/journal.pone.0070264 (PMC3729555; doi:10.1371/journal.pone.0070264)
Supplement: File S1 — Figure S1. SBDTs at (A) TFM and (B) BPW with borehole flow regimes and packer intervals in this study. Times refer to time (h: hours, d: days) after dilution and B is background; RWL is rest water level; U, M, L are upper, middle and lower intervals, respectively. Table S1. All hydrochemical data. Note: Number in interval name refers to when sample was taken during pumping; all forms of phosphate and nitrogen are total concentrations. Table S2. Sizes of whole captured invertebrates with pumped volume. (ZIP) [file pone.0070264.s001.zip › Table S2.docx]

| **Interval** | **Volume pumped (m^3^)** | **Size (mm)** |
| --- | --- | --- |
| BPW Middle | 0.25 | 1.2 |
| BPW Middle | 1 | 1.7 |
| BPW Middle | 1.5 | 1.6 |
| BPW Middle | 2 | 1.6 |
| BPW Middle | 3.5 | 1.1 |
| BPW Middle | 3.5 | 1.7 |
| BPW Middle | 5 | 1.3 |
| BPW Lower | 0.25 | 1.7 |
| BPW Lower | 0.25 | 1.1 |
| BPW Lower | 3 | 1.1 |
| TFM Middle | 1 | 1.1 |
| TFM Middle | 3 | 1.1 |
| TFM Middle | 4 | 1.4 |
| TFM Middle | 4 | 1.4 |
| TFM Middle | 5 | 1.5 |
| TFM Lower | 0.1 | 1.1 |
| TFM Lower | 0.5 | 1.1 |
