# Supplementary material for: Using Boreholes as Windows into Groundwater Ecosystems
Source: PLoS One. 2013 Jul 31;8(7):e70264. doi: 10.1371/journal.pone.0070264 (PMC3729555; doi:10.1371/journal.pone.0070264)
Supplement: File S1 — Figure S1. SBDTs at (A) TFM and (B) BPW with borehole flow regimes and packer intervals in this study. Times refer to time (h: hours, d: days) after dilution and B is background; RWL is rest water level; U, M, L are upper, middle and lower intervals, respectively. Table S1. All hydrochemical data. Note: Number in interval name refers to when sample was taken during pumping; all forms of phosphate and nitrogen are total concentrations. Table S2. Sizes of whole captured invertebrates with pumped volume. (ZIP) [file pone.0070264.s001.zip › Table S1.docx]

| **Interval** | **Temp** | **pH** | **DO** | **SEC** | **HCO_3_** | **SRP** | **TDP** | **TP** | **DHP** | **DOC** | **F** | **Cl** | **NO_2_** | **Br** | **NO_3_** | **SO_4_** | **Na** | **K** | **Ca** | **Mg** | **B** | **Fe** | **Mn** | **Zn** | **Cu** | **Al** |
| --- | --- | --- | --- | --- | --- | --- | --- | --- | --- | --- | --- | --- | --- | --- | --- | --- | --- | --- | --- | --- | --- | --- | --- | --- | --- | --- |
|  | **(oC)** |  | **mg/l** | **mS/cm** | **mg/l** | **μg/l** | **μg/l** | **μg/l** | **μg/l** | **mg/l** | **mg/l** | **mg/l** | **mg/l** | **mg/l** | **mg/l** | **mg/l** | **mg/l** | **mg/l** | **mg/l** | **mg/l** | **μg/l** | **μg/l** | **μg/l** | **μg/l** | **μg/l** | **μg/l** |
| **BPW** |  |  |  |  |  |  |  |  |  |  |  |  |  |  |  |  |  |  |  |  |  |  |  |  |  |  |
| Upper <0.02 | 10.0 | 7.1 | 8.8 | 782 | 300 | 26 | 74 | 129 | 48 | 7.3 | 0.17 | 42.0 | 0.05 | 0.06 | 43.9 | 21.8 | 22.3 | 2.0 | 117.0 | 2.7 | 24.7 | 406.5 | 62.8 | 727.9 | 17.1 | 15.7 |
| Upper 2.5 | 10.1 | 7.4 | 8.4 | 679 | 302 | 17 | 28 | 63 | 11 | 1.5 | 0.13 | 18.4 | 0.00 | 0.05 | 36.4 | 10.1 | 8.7 | 1.3 | 112.8 | 1.7 | 16.1 | 7.1 | 0.9 | 23.7 | 1.1 | 4.5 |
| Upper 5.0 | 10.1 | 7.4 | 8.4 | 663 | 301 | 2 | 58 | 75 | 56 | 2.1 | 0.13 | 18.4 | 0.00 | 0.04 | 36.6 | 10.1 | 8.8 | 1.3 | 113.0 | 1.7 | 16.1 | 5.1 | 0.5 | 16.1 | 0.4 | 7.3 |
| Middle 0.02 | 10.1 | 7.3 | 8.1 | 650 | 297 | 2 | 35 | 1016 | 33 | 2.5 | 0.14 | 18.6 | 0.00 | 0.04 | 36.6 | 10.5 | 9.0 | 3.9 | 111.1 | 1.7 | 16.8 | 6.0 | 7.6 | 169.2 | 2.6 | NA |
| Middle 2.5 | 10.2 | 7.3 | 7.6 | 631 | 302 | 16 | 41 | 152 | 25 | 1.0 | 0.13 | 17.9 | 0.00 | 0.00 | 35.7 | 9.9 | 8.8 | 1.6 | 110.7 | 1.7 | 14.9 | 11.2 | 1.3 | 23.9 | 1.0 | 7.5 |
| Middle 5.0 | 10.0 | 7.3 | 8.3 | 619 | 309 | 17 | 36 | 178 | 19 | 1.0 | 0.15 | 18.0 | 0.00 | 0.04 | 35.1 | 10.1 | 8.8 | 1.3 | 111.3 | 1.8 | 15.4 | 8.3 | 1.7 | 23.3 | 0.2 | 6.1 |
| Lower 0.02 | 9.6 | 6.7 | 8.8 | 637 | NA | 4 | 28 | 289 | 24 | 2.2 | 0.14 | 21.1 | 0.03 | 0.04 | 37.1 | 11.0 | 11.0 | 1.5 | 115.3 | 1.8 | 17.6 | 30.2 | 15.6 | 237.0 | 3.5 | 11.0 |
| Lower 2.5 | 10.1 | 7.3 | 9.3 | 613 | NA | 6 | 26 | 30 | 20 | 1.1 | 0.12 | 18.7 | 0.00 | 0.00 | 36.9 | 10.4 | 8.9 | 1.8 | 110.7 | 1.7 | 16.3 | 3.0 | 1.2 | 20.9 | 1.3 | 5.9 |
| Lower 5.0 | 10.0 | 7.4 | 9.8 | 609 | NA | 14 | 23 | 27 | 9 | 0.8 | 0.13 | 18.4 | 0.00 | 0.04 | 36.8 | 10.2 | 8.8 | 1.4 | 112.4 | 1.7 | 16.4 | 3.0 | 0.3 | 16.7 | 0.3 | NA |
| **TFM** |  |  |  |  |  |  |  |  |  |  |  |  |  |  |  |  |  |  |  |  |  |  |  |  |  |  |
| Upper <0.02 | 12.0 | 7.0 | 6.9 | 579 | 293 | 7 | 27 | 153 | 20 | 2.6 | 0.13 | 16.7 | 0.00 | 0.04 | 44.8 | 12.4 | 7.3 | 1.26 | 112.5 | 1.57 | 12.7 | 56.9 | 23.1 | 421.8 | 3.2 | 53.1 |
| Upper 2.5 | 11.1 | 7.1 | 6.7 | 564 | 278 | 10 | 26 | 41 | 16 | 0.8 | 0.13 | 15.3 | 0.00 | 0.04 | 39.6 | 9.6 | 6.7 | 1.08 | 107.8 | 1.45 | 10.7 | 14.3 | 2.3 | 26.0 | 0.3 | 14.7 |
| Upper 5 | 11.1 | 7.2 | 7.0 | 574 | 275 | 3 | 23 | 26 | 20 | 1.0 | 0.12 | 15.8 | 0.00 | 0.04 | 40.2 | 9.7 | 7.0 | 1.23 | 108.6 | 1.46 | 10.6 | 7.3 | 1.7 | 28.4 | 0.5 | 11.7 |
| Middle <0.02 | 14.4 | 7.0 | 8.9 | 629 | 285 | 4 | 31 | 35 | 27 | 0.9 | 0.13 | 16.3 | 0.00 | 0.03 | 43.6 | 11.3 | 7.0 | 1.11 | 115.1 | 1.50 | 11.0 | 24.1 | 9.8 | 96.8 | 1.9 | 13.2 |
| Middle 2.5 | 13.1 | 6.9 | 7.9 | 614 | 278 | 18 | 21 | 22 | 3 | 1.5 | 0.14 | 16.0 | 0.00 | 0.03 | 39.3 | 9.6 | 7.1 | 1.22 | 107.2 | 1.48 | 10.8 | 3.1 | 1.9 | 56.4 | 0.6 | 3.0 |
| Middle 5.0 | 11.4 | 7.2 | 8.0 | 587 | 278 | 19 | 22 | 23 | 3 | 0.0 | 0.12 | 15.3 | 0.00 | 0.03 | 39.1 | 9.6 | 6.9 | 1.13 | 105.3 | 1.48 | 10.9 | 1.9 | 2.5 | 35.6 | NA | NA |
| Lower <0.02 | 18.3 | 7.1 | 9.2 | 659 | 300 | 4 | 27 | 63 | 23 | 1.9 | 0.11 | 16.8 | 0.00 | 0.03 | 42.4 | 10.9 | 7.4 | 1.28 | 108.2 | 1.51 | 11.3 | 2.2 | 8.1 | 114.4 | 1.4 | 9.6 |
| Lower 2.5 | 12.3 | 7.1 | 7.6 | 629 | 283 | 13 | 20 | 51 | 7 | 1.1 | 0.13 | 16.1 | 0.00 | 0.04 | 33.4 | 8.1 | 7.7 | 1.51 | 103.9 | 1.67 | 11.9 | 34.1 | 1.4 | 58.9 | 0.0 | 65.9 |
| Lower 5.0 | 12.2 | 7.3 | 7.5 | 610 | 283 | 16 | 21 | 31 | 5 | 0.9 | 0.14 | 15.5 | 0.00 | 0.03 | 34.3 | 8.3 | 7.5 | 1.41 | 106.0 | 1.64 | 11.7 | 8.5 | 0.4 | 33.0 | NA | 20.3 |
