# Supplementary figures and images for: Using Boreholes as Windows into Groundwater Ecosystems
Source: PLoS One. 2013 Jul 31;8(7):e70264. doi: 10.1371/journal.pone.0070264 (PMC3729555; doi:10.1371/journal.pone.0070264)

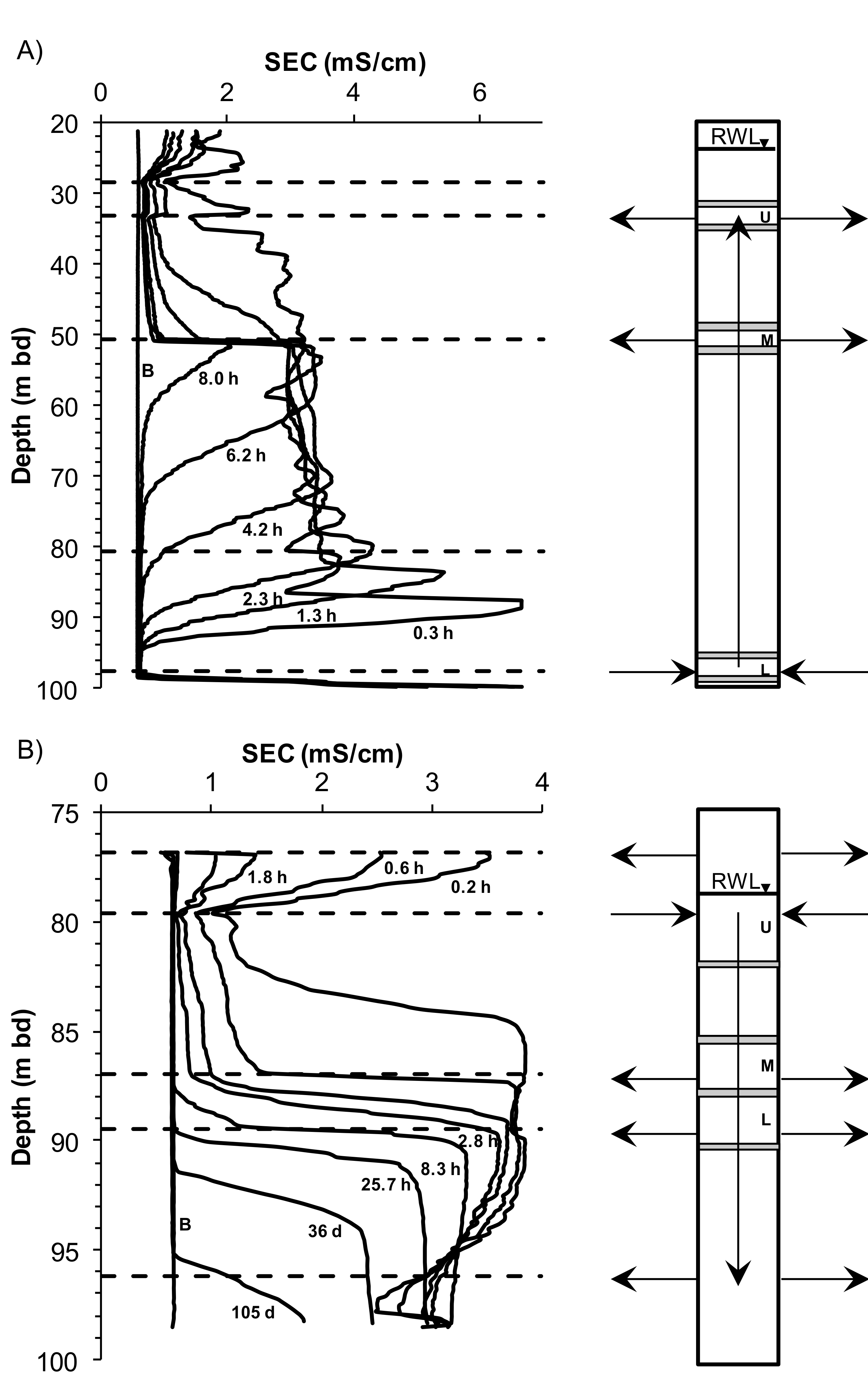

Supplement: File S1 — Figure S1. SBDTs at (A) TFM and (B) BPW with borehole flow regimes and packer intervals in this study. Times refer to time (h: hours, d: days) after dilution and B is background; RWL is rest water level; U, M, L are upper, middle and lower intervals, respectively. Table S1. All hydrochemical data. Note: Number in interval name refers to when sample was taken during pumping; all forms of phosphate and nitrogen are total concentrations. Table S2. Sizes of whole captured invertebrates with pumped volume. (ZIP) [file pone.0070264.s001.zip › Figure S1.tif]
